# Selective under-representation of Pacific peoples in population estimates for health indicator measurements in Aotearoa New Zealand misinforms policy making

**DOI:** 10.1186/s12889-024-17984-2

**Published:** 2024-02-22

**Authors:** Gerard J.B Sonder, Corina Grey, Debbie Ryan, Jacqueline Cumming, Andrew Sporle, Philip C. Hill

**Affiliations:** 1 Pacific Perspectives Ltd, PO Box 8010, Wellington, New Zealand; 2 Department of Internal Medicine, Amsterdam Infection & Immunity Institute (AIII), Amsterdam UMC, location Academic Medical Center, Amsterdam, the Netherlands; 3 Department of General Practice and Primary Health Care, School of Population Health, University of Auckland, Auckland, New Zealand; 4 iNZight Analytics Ltd, Auckland, New Zealand; 5 Department of Statistics, The University of Auckland, Auckland, New Zealand; 6 Centre for International Health, Division of Health Sciences, University of Otago, Dunedin, New Zealand

**Keywords:** Pacific, Population, Statistics, Institutional, Racism, Health

## Abstract

**Background:**

The Census of Populations and Dwellings’ is the five yearly population count of Aotearoa New Zealand. Best available populations (BAP) are subnational projections based on census data and demographic assumptions developed for healthcare planning and funding allocation but are also used as the denominator for health indicator monitoring. Pacific people are systematically undercounted, but the impact on health statistics is not well studied. For COVID-19 vaccination coverage, health service user (HSU) data were considered a more reliable denominator than BAP but introduced new biases. We aimed to understand how the choice of denominator population impacts estimates of population size and health system performance for Pacific people at a local level.

**Methods:**

We described how declining census response rates affected population data quality. We compared BAP and HSU data at district level. For the indicators ‘access to primary care’ and ‘cervical cancer screening uptake’ we replaced currently used BAP denominators with HSU and examined the impact for different ethnic groups in different geographic districts.

**Results:**

Overall Census 2018 response declined by 10%, but for Māori and Pacific people by 21% and 23%, respectively. This inequitably affected BAP accuracy. Census undercount was highest in the district with the largest Pacific populations, where HSU exceeded BAP most. Notably, ‘access to primary care’ for Pacific people in this district consistently exceeds 100%. Using BAP, both health indicators are currently estimated as *highest* for Pacific people compared to other ethnic groups, but when based on HSU, they dropped to *lowest*. Similar, but less pronounced trends occurred in other districts. Changes in trends over time for both indicators coincided mostly with adjustments in BAP, rather than changes in the numerators.

**Conclusions:**

The current use of BAP denominators for health statistics does not enable reliable monitoring of key health indicators for Pacific people. HSU denominators are also unsuitable for monitoring health. Exploring the feasibility of a real-time population register is strongly recommended as a new, transparent, way of obtaining more reliable, timely population data to guide policymaking and underpin a more equitable health system under the health reforms. Meanwhile, reporting of ethnic specific outcomes need to include a clear assessment of the potential for bias due to inaccurate population estimates.

**Supplementary Information:**

The online version contains supplementary material available at 10.1186/s12889-024-17984-2.

## Introduction

In Aotearoa New Zealand (ANZ), the indigenous Māori population and Pacific peoples (the fourth largest ethnic group when considered as a single population group) have significantly poorer health status than people from other ethnic groups [1–3]. Improving equity in health status between population groups has been a strategic priority in successive health system reforms. In 2020, a comprehensive review of the health and disability system concluded that there were still major – and sometimes increasing - disparities in health status between ethnic groups, and that inequities differed between health district (District Health Board (DHB)) areas [4] This resulted in another major health reform, through the Pae Ora (Healthy Futures) Act 2022 [5], which took effect on 1 July 2022. The local DHBs were disestablished and replaced with the national organisations ‘Te Whatu Ora’ (Health New Zealand (HNZ) [6], ‘Te Aka Whai Ora’(the Māori Health Authority to drive improvement in Māori health) [7]; a new Public Health Agency (within the Ministry of Health) [8], and a centralised National Public Health Service (within Te Whatu Ora) instead of the previous 12 Public Health Units [9]. Instead of the 20 DHBs, 60–80 smaller ‘Localities’ are to be established, [10] which are geographic areas that are home to communities with their own specific health needs. A population health approach will be embedded in these localities for better implementation of (public) health measures. A new Pacific Health Strategy has also been developed to support Pacific peoples’ health [11].

Whilst there has been attention to emerging evidence about disparities in health status for Pacific populations over the past 20 years, and several Pacific Health Action Plans have been developed [11, 12], inconsistent reporting and lack of visibility of Pacific ethnic groups in health data and statistics prevent a clear understanding of the health issues Pacific people face, complicating policy making. Underrepresentation, specifically of Pacific people in population statistics, is well documented [13], but attention to this issue has usually only occurred when health statistics exceed estimates of 100%, exposing inaccurate denominators. Health system reports often include a caveat that data should be ‘interpreted with care’, without addressing the possible impacts of these inaccuracies [14, 15].

Having accurate population data is a prerequisite for making informed decisions and promoting equitable health in populations and communities [16]. High-quality census data collected as part of the official five-yearly count of people and dwellings is required for accurate population estimates [17]. During the rollout of COVID-19 vaccinations, media interest and public reporting of vaccination coverage led to the Ministry of Health and Statistics New Zealand (StatsNZ) announcing - without much public explanation - that Health Service User data (HSU2020) would provide more reliable estimates of COVID-19 vaccination coverage than the generally used population projections or “best available population” (BAP) measure based on census data [18]. The impact of the introduction of new biases when using HSU [19], while continuing use of BAP denominators in other health estimates, have been overlooked.

In this article, we aimed to understand how the choice of denominator population affects estimates of population size and health system performance for Pacific peoples at a local level by describing and analysing the quality and appropriateness of the two denominators currently used in health statistics (HSU and BAP) using two indicators as examples. Furthermore, we discuss how the use of population estimates in other health statistics may also contribute to persistent institutional racism experienced by Pacific people.

## Methods

This descriptive study followed the ‘Strengthening the Reporting of Observational Studies in Epidemiology’ (STROBE) reporting guideline [20]. We used only publicly available data; therefore, the study was exempt from ethics review in accordance with the New Zealand Operational Standard for Ethics Committees [21].

First, we describe how the declining census response rates have led to disproportional falls in the accuracy and quality of data for Māori and Pacific populations. Second, we investigate the interaction between geographic area and ethnicity by comparing published BAP and HSU population data at a district level by making comparisons between the Counties Manukau district (the area with the largest Pacific population in ANZ) and all other districts combined. Third, for both ‘access to primary health care’ and ‘uptake of cervical screening’ indicators, we replace BAP denominators with HSU denominators and examine the different impact this has on estimates for different ethnic groups and in the different geographic areas. Finally, we look at the impact on indicator trends of interim methodology adjustments for estimating BAP.

### Data sources

#### Census population estimates (Estimated Residence Population ERP and Projected Resident Population PRP)

The StatsNZ ‘Census of Populations and Dwellings’ [22] collects data from all people in ANZ using standardised questionnaires [23, 24], including their residential address and self-identified ethnicity. People can belong to more than one ethnic group and information is collected in a standardised manner, so that responses can be coded to specific group categories at the most detailed level [25]. Official population statistics and methods are summarised in Table 1. A post-enumeration survey (PES) is held after census night, to estimate how many residents were missed. Following each census, national population *estimates* (Estimated Resident Population, ERP) are produced quarterly based on registered births, deaths, and net migration. Between census dates, the ERP provides the best measure of the usually resident population of ANZ [26].

**Table 1:** Components used to estimate the key population statistics: usually resident population; Estimated resident Population (ERP) and Projected Resident Population (PRP), produced by stats NZ

	Census Usually Resident Population^a^	Estimated Resident Population (ERP)	Projected Resident Population (PRP)^b^
**Counted by Census**	Residents of that area in the area on census night	Residents of that area in the area on census night	Residents of that area in the area on census night
	Residents of that area elsewhere in NZ on census night	Residents of that area elsewhere in NZ on census night	Residents of that area elsewhere in NZ on census night
**Estimated from Post-enumeration Survey (PES)**	-	Residents missed by census (net census undercount)	Residents missed by census (net census undercount)
**Estimated from administrative data**	*Census 2018: Residents missing in census response but active in tax, health, education, and ACC (Accident Compensation Corporation) in the past 2 years.*	Residents temporarily overseas on census night	Residents temporarily overseas on census night
		Births, deaths, and net migration since census night	*Assumptions* about *future* birth rates, death rates and net migration

Source: Tatauranga Aotearoa Stats NZ: Population Statistics – user guide. https://www.stats.govt.nz/methods/population-statistics-user-guide

^a^Because of the low response rate of Census 2018, residents who had not responded but had been active in one of these administrative data in the previous 2 years, were added to the Census 2018 enumerations

^b^These national projections are published annually by StatsNZ

Subnational population *projections* (Projected Resident Population, PRP) are produced annually, modelled on assumptions about future birth rates, death rates and net migration. Because internal migrants are not required to register changes in address with any agency, internal migration is the most difficult component of net migration’s contribution to subnational population estimations [27]. The census question “usual residence five years ago” has been the authoritative data source for measuring internal migration.

Both estimates and projections remain provisional for up to two years and are revised to incorporate external and internal migration estimates. Subnational projections do not include projections by health district [28].

### Best Available Population projections used in health statistics (BAP)

Population demographic information is used at a local health district level by health providers for service planning and delivery. To facilitate access to updated estimates, health entities publish more detailed subnational population projections that include age, gender, and prioritised ethnicity following the prioritisation order: ‘Māori’, ‘Pacific’, ‘Asian’ and ‘Other’ (i.e., all those who identify as Māori and/or another ethnicity are counted as Māori, those who identify as Pacific but not Māori are counted as Pacific, etc.) [29]. These provisional data are not considered official StatsNZ statistics and can be adjusted at any time (without following a regular publication schedule). These projections use census population estimates as a starting point and are known as BAP. Te Whatu Ora - Health New Zealand is now responsible for publishing these estimates, and they are frequently used as the denominator in health statistics [18].

### Health Service User data (HSU)

The HSU population is defined as an estimate of the population using health services ‘*within a specific time period’* [30]. People are included in the HSU dataset if they have used publicly funded health services (including births and deaths) or enrolled in a Primary Healthcare Organisation (PHO). Births are included if the birth was in a hospital, attended by a Lead Maternity Carer, or registered with the Department of Internal Affairs. Exit from the HSU population occurs through death, lapsed PHO enrolment, or ‘inactivity of health service use’. HSU is a subset of the National Health Index (NHI) database that was established to provide a mechanism for identifying every health care user by assigning each user a unique NHI number [30]. Demographic variables, including age, gender, and prioritised ethnicity in the HSU dataset, are derived from the NHI. Area of residence is derived from the latest address registration [31].

HSU is calculated six months after the end of the reference period, reflecting the lag needed for the data used to construct the HSU to be sufficiently complete [25]. HSU data are not published regularly, they can be updated retrospectively, and are not considered official statistics.

### Primary Healthcare Organisation (PHO) enrolment data

In ANZ, people choose their own primary health care provider or General Practice (GP) and enrolment is voluntary [15]. Benefits of enrolment with a GP include lower costs for consultations and prescriptions, and access to health programmes such as reminders for vaccinations and cancer screening. GPs receive capitation funding based on the socio-demographic characteristics of their enrolled patients. Enrolment demographics have been collected in real-time through a National Enrolment Service since 2019 [32]. People are unenrolled when they enrol with another PHO (so there should be no duplication) or automatically, when they have not used their primary care provider in three years.

PHO enrolment has been published quarterly on the Ministry of Health website since 2019 [33]. The ‘access to primary care’ indicator was defined as the percentage of enrolled patients estimated by prioritised ethnic group, age, socioeconomic deprivation index and district, using BAP as denominator. Prioritisation of ethnicity data is carried out so that people identifying with multiple ethnic groups are prioritised to a single response, in the following prioritisation order: ‘Māori’, ‘Pacific’, ‘European and Other’ [33].

### Coverage of the National Cervical Cancer Screening programme

From 2019 up until September 2023, the National Cervical Screening Programme in ANZ has recalled women and people with a cervix or a vagina between 25 and 70 years who have ever been sexually active every three years for smear tests (prior to 2019, screening commenced at 20 years, and an HPV test has been offered instead since September 2023) [34]. The National Screening Unit publishes three-yearly national coverage reports in an interactive tool [35]. Coverage is reported by four main prioritised ethnic groups (‘Māori’, ‘Pacific’, ‘Asian’ and ‘Other’) and by district. The most recent denominator used for these reports is ‘the 2021 population update of the 2018 Census’ (which is a version of BAP) [18]. How this BAP denominator is translated into the estimated *eligible* population, or whether the 2021 adjustments to these BAP projections have been retrospectively implemented in these estimates is not stated.

### Analysis

For our comparison of HSU data with BAP population projections, we used the HSU data that were published as the denominators for COVID-19 vaccination coverage as part of Ministry of Health publications in 2020 and 2021 [36] and extracted BAP data from the webtool [18]. For the ‘access to primary care’ indicator, we collated PHO enrolment data (based on BAP) between quarter 1 2019 (1 January − 31 March 2019) and quarter 3 2022 (1 July 2022-31 September 2022) [33] to study trends for different ethnic groups and compared results for 2020 and 2021 using HSU denominators. For the cervical cancer screening indicator, we extracted three-year cervical cancer screening coverage data for each year ending December between 2008 and 2022 by district and ethnic group. We plotted total screening numbers as well as coverage rates based on eligible women as a proportion of BAP denominators between 2008 and 2022 and selected the female population aged 25–69 years from HSU2020 and HSU2021 databases as the eligible population to compare this coverage between the two denominators.

## Results

### Census 2018 population, Estimated Resident Population (ERP), Projected Resident Population (PRP)

**Fig. 1:**
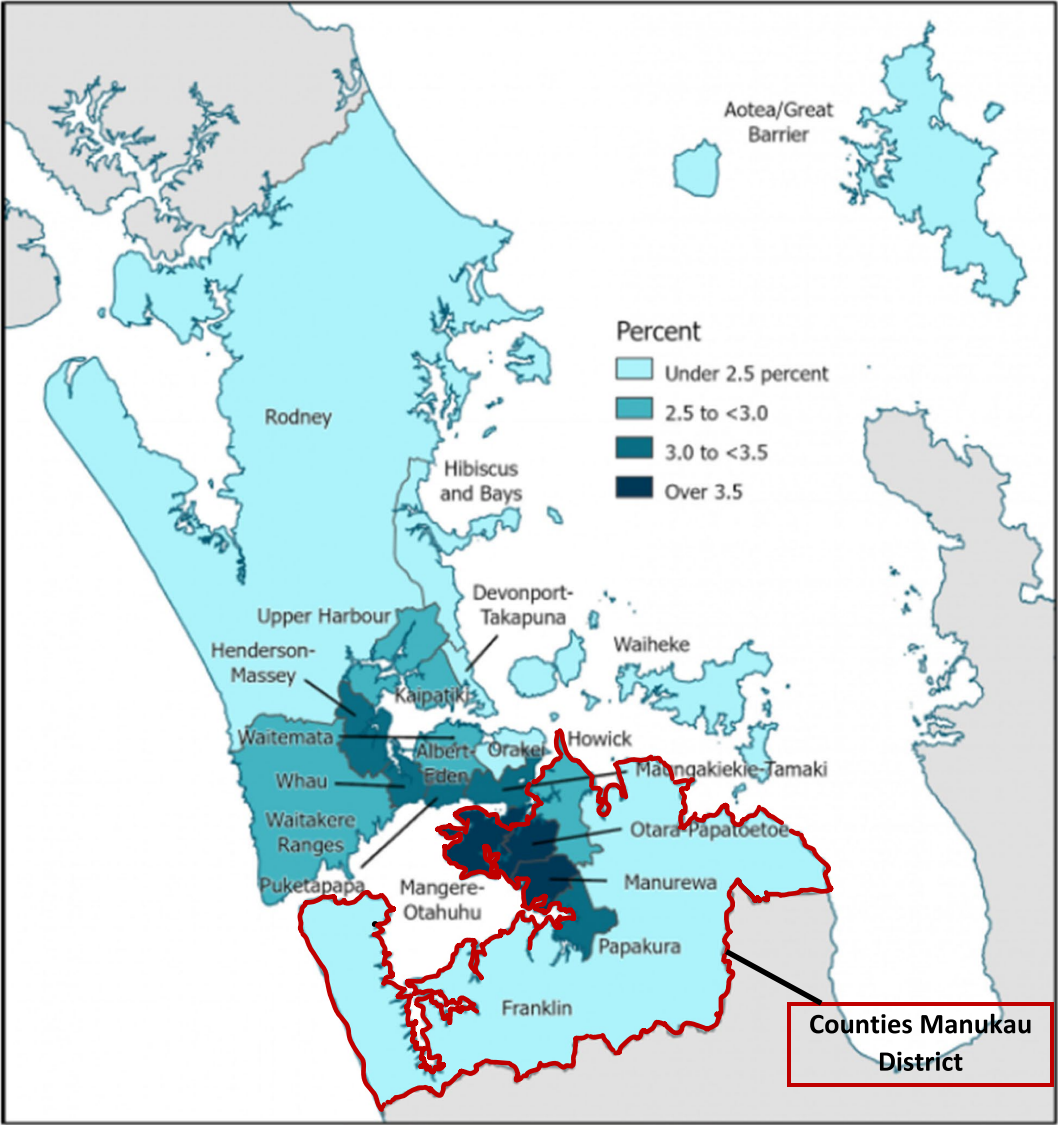
Geographic coverage of Census 2018 in Auckland Local Board Areas, expressed as percentage median undercount estimate, based on Post-Enumeration Survey Census 2018. Source: Tatauranga Aotearoa Stats NZ. Post-enumeration survey 2018: Methods and results. https://www.stats.govt.nz/methods/post-enumeration-survey-2018-methods-and-results/

Census 2018 was the first ‘digital-first’ census undertaken in ANZ [37]. The overall response rate of 82% (Table 2), was 10% lower for the total population than the previous census but 21% lower for Māori (89–68%) and 23% lower for Pacific people (88–65%) [38]. To address this disparity, post-hoc, collated administrative data from the Integrated Data Infrastructure (IDI) [39], a large research database from different government sectors, was used to add people that were identified as missing in the Census 2018 but who were included in the taxation, health, education, or Accident Compensation Corporation datasets in the previous two years. (Tables 1 and 2). Administration data, however, do not meet the accuracy requirements for producing official statistics: the collection methods are not standardised, are highly variable, and usually not reported. For example, data on *ethnicity* are usually not collected in a standardised manner and information about when the *usual residential address* was last updated is not collected. The post enumeration survey results showed that groups with lower participation in the census were males, young adults, and people born overseas and, of all ethnic groups, the undercount for Pacific people was the highest (Table 2). Geographically, the undercount was highest in the Counties Manukau District local board areas of Ōtara-Papatoetoe, Manurewa and Mangere-Otahuhu (Fig. 1).

**Table 2:** Census 2018 usually resident population and Estimated Resident Population (ERP), with key population statistics, imputed administrative enumerations and estimated undercount by non-prioritised ethnic group. All percentages are relative to the ERP

Source	Māori	Pacific	Non-Māori Non-Pacific	Total
**Individual form**^**a**^ **(response rate)**^**b**^	551,000 (68%)	259,000 (65%)	3,613,000 (86%)	4,423,000 (82%)
**Household form**^**c**^	49,000 (6%)	33,000 (8%)	151,000 (4%)	233,000 (4%)
**Admin enumerations**^**d**^	176,000 (22%)	90,000 (22%)	351,000 (8%)	617,000 (11%)
**Census Usually Resident Population**	**776,000 (96%)**	**382,000 (95%)**	**4,116,000 (98%)**	**5,274,000 (97%)**
**Estimated Post Enumeration Survey (PES) undercount**^**e**^	35,000 (4%)	20,000 (5%)	87,000 (2%)	142,000 (3%)
**Estimated Resident Population (ERP)**	**811,000 (100%)**	**401,000 (100%)**	**4,203,000 (100%)**	**5,415,000 (100%)**^f^

Source: Tatauranga Aotearoa StatsNZ. Post-enumeration survey 2018: Methods and results. https://www.stats.govt.nz/methods/post-enumeration-survey-2018-methods-and-results/

^a^Only from individuals who filled out an individual form, standardised uniform information on ethnicity is collected

^b^The *response rate* is the number of individuals filling out an individual form divided by the ERP

^c^Individuals who did not fill out an individual form but were on a household listing only. These individuals are not asked to which ethnic group they belong

^d^Before 2018, administrative data was used to impute missing data on census forms. In 2018, because of the low response rates, people who were missing in census response were included if they had been active in tax, health, education, and ACC (Accident Compensation Corporation) in the past 2 years. Admin data are not collected in a standardised manner

^e^Estimated census undercount based on Post-Enumeration Survey

^f^ERP is higher than the population total, since ERP is reported by total ethnicity (instead of prioritised ethnicity)

### Comparison of HSU and BAP denominators

The denominator first used to estimate COVID-19 vaccination coverage was HSU2020 (Table 3). Although National Immunisation Programme vaccinations are considered Health Service Use and are included in the HSU dataset, COVID-19 vaccinations were initially not included in the HSU2020 dataset. Therefore, people who were not enrolled with a PHO or whose only use of health care was a COVID-19 vaccination were represented in the numerator, but not in the denominator. To overcome this discrepancy, a new, ‘hybrid’ 2020/2021HSU was created, covering a reference period of 22 months (1 January 2020 to 31 October 2021) that included ‘COVID-19 vaccine only’.

**Table 3:** Summary of differences in ethnicity between Projected Resident Populations (PRP) based on Census 2018, and Health Service User populations (HSU), relative to HSU, by year in 2020 and 2021, respectively

	2020				2021			
Ethnic group, by order of prioritisation	PRP^a^ 2020	HSU 2020^b^	Difference HSU2020-PRP2020 #	Difference %	PRP^a^ 2021	HSU 2021^c^	Difference HSU2021-PRP2021#	Difference %
Māori	854,900	776,200	78,200	10%	875,400	806,200	69,200	9%
Pacific	346,600	371,600	-25,000	-7%	352,200	397,200	-45,000	-11%
Asian	840,300	741,400	98,900	13%	848,800	844,800	4,000	0%
Other^d^	3,048,400	3,134,500	-86,100	-3%	3,046,200	3,220,700	-174,500	-5%
Unspecified	0	23,000	23,000	-100%	0	44.900	-44,900	-100%
Total population	5,090,200	5,046,700	43,500	1%	5,122,600	5,313,800	-191,200	-4%

Source: StatsNZ: Review of Health Service User population methodology 04 August 2022 https://www.stats.govt.nz/reports/review-of-health-service-user-population-methodology/

^a^PRP = Projection of the Resident Population based on Census 2018

^b^HSU 2020 = Health Service User population comprising people who were enrolled with a Primary Healthcare Organisation or used healthcare between 1 January 2020 and 31 January 2020. People who were not enrolled and were only vaccinated for COVID-19 without any other health care use were not included. https://www.stats.govt.nz/reports/review-of-health-service-user-population-methodology/

^c^HSU 2021 = Health Service User population comprises people who were enrolled with a Primary Healthcare Organisation or used healthcare between 1 January 2020 and 31 October 2021. People who were not enrolled and were only vaccinated for COVID-19 without any other health care use were included as well. https://www.stats.govt.nz/reports/review-of-health-service-user-population-methodology/

^d^Middle Eastern, Latin American, African, European, and Other (including New Zealander)

In the post-hoc review following introduction of HSU denominators, StatsNZ compared 4 different denominators: ‘2020HSU’, 2020/2021HSU’, ‘PRP2020’ and ‘PRP2021’ [31] and found that particularly for Pacific people, HSU data substantially exceeded the projected population, both in 2020 (25,000 people, 7%) and in 2021 (45,000 people, 11%) (Table 3). These differences are explained by underestimates in the projected populations. Discrepancies between both databases were not equally distributed amongst different age groups, genders, ethnic groups, or districts [31].

To investigate how these substantial differences for Pacific people were distributed across the country, we plotted HSU2020 and HSU2020/2021, published by the Ministry of Health as denominators for COVID-19 vaccination coverage [36], along with BAP2020 and BAP2021 [18] in one table by ethnicity, gender, and district (Supplementary Table 1).

Instead of the StatsNZ estimates of 25,000 and 45,000 additional Pacific people (Table 3), we found only 11,611 and 26,966 more Pacific people in the HSU dataset compared with BAP in 2020 and 2021, respectively (Supplementary Table 1). Comparing denominators at district level showed that the largest BAP undercounts were consistently in the Pacific populations in Counties Manukau district.

### PHO enrolment and access to primary care

**Fig. 2:**
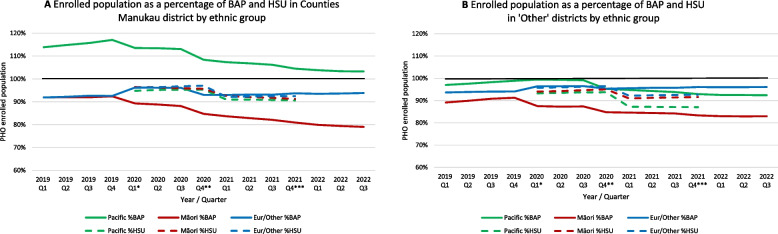
Trends in PHO enrolment by ethnic group based on BAP and HSU denominators, respectively, in Counties Manukau (**2A**) and other Districts (**2B**), 2019–2022. PHO = Primary Healthcare Organisation; BAP = Best Available Population, projections based on census data; Q = quarter; Eur/Other = New Zealand European and other ethnic groups. *Interim adjustment in BAP. The updated projections use the latest subnational population estimates and updated methodology to calculate migration. The combined impact is a large variation in projected population for some DHBs when comparing to previous releases and this is most noticeable in Auckland. **Interim adjustment in BAP. Population is based on projections provided by Stats NZ in Dec 2020. ***Interim adjustment in BAP. Population is based on projections provided by Stats NZ in Dec 2021

**Fig. 3:**
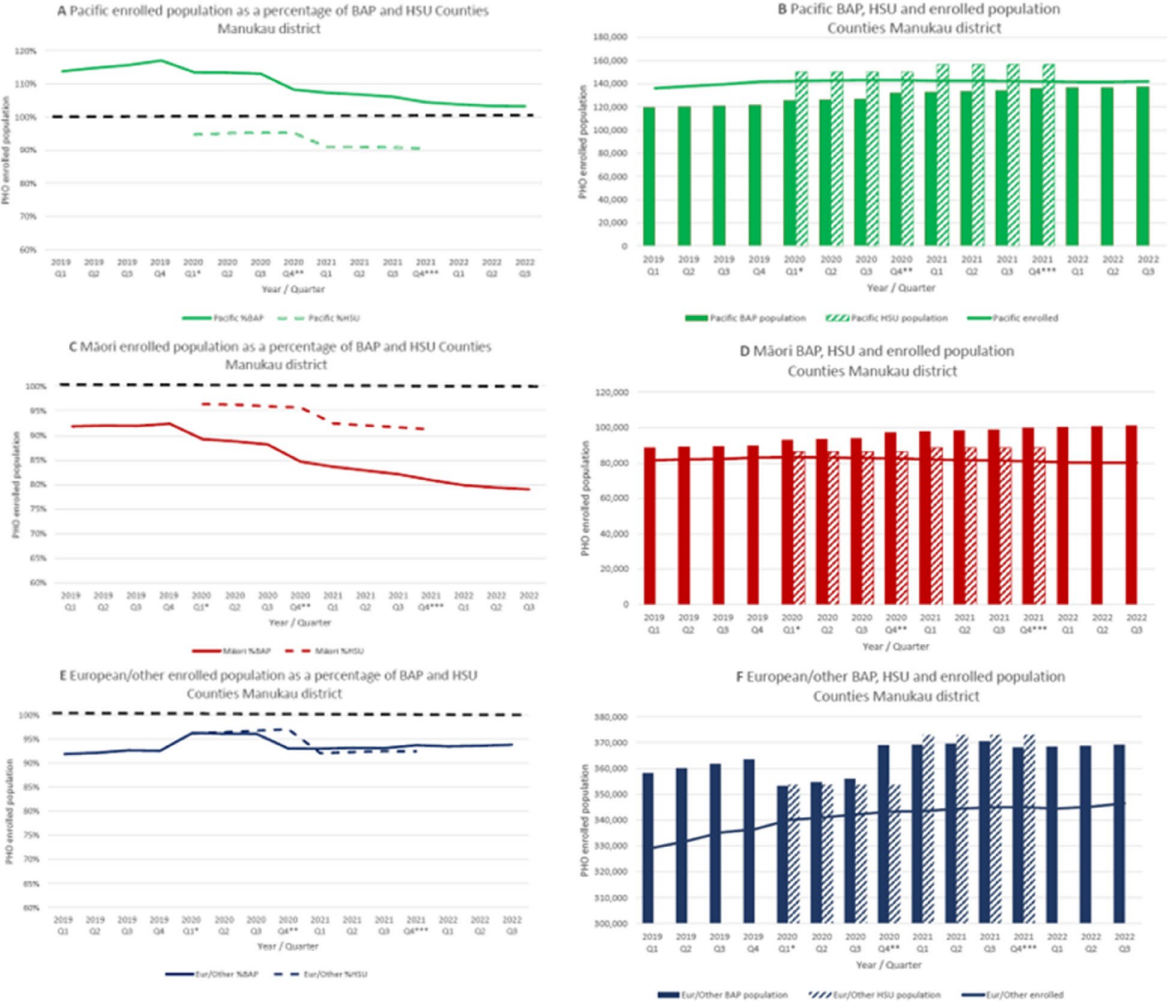
Trends in PHO enrolment percentage based on BAP and HSU denominators (**3A**, **3C**, **3E**) compared to trends in enrolment (numerator) and estimated populations (denominators) (**3B**, **3D**, **3F**) by ethnic group in Counties Manukau District, 2019–2022. PHO = Primary Healthcare Organisation; BAP = Best Available Population, projections based on census data; Q = quarter; Eur/Other = New Zealand European and other ethnic groups. - - - - - 100% line. *Interim adjustment in BAP. The updated projections use the latest subnational population estimates and updated methodology to calculate migration. The combined impact is a large variation in projected population for some DHBs when comparing to previous releases and this is most noticeable in Auckland. **Interim adjustment in BAP. Population is based on projections provided by Stats NZ in Dec 2020. ***Interim adjustment in BAP. Population is based on projections provided by Stats NZ in Dec 2021

Quarterly trends in PHO enrolment by ethnicity between 2019 and 2022 are shown in Fig. 2A (Counties Manukau District) and Fig. 2B (Other Districts), as published based on BAP [33] and HSU (Supplementary Table 1) denominators. In Counties Manukau, enrolment coverage for Pacific and Māori based on BAP steadily declined from 117 to 103% for Pacific, and from 92 to 79% for Māori. For the ‘Other’ ethnic group, enrolment coverage increased from 92 to 94%. Replacing BAP with HSU denominators had the largest effect on Pacific estimates, that no longer exceeded 100%, and were now lowest of all ethnic groups. Māori coverage estimates were higher with HSU then with BAP denominators, whereas the difference for ‘Other’ ethnic groups was smallest. The numerators and denominators (Fig. 3) used for these estimates by ethnicity show that for both Pacific and ‘Other’ ethnic groups, the enrolled numbers steadily increased each quarter, whereas for Māori the enrolled population steady declined since the first quarter of 2020. Changes in enrolment coverages mostly coincided with changes in BAP methodology, or with a major adjustment in HSU methodology (i.e., inclusion of ‘COVID-19 vaccinations only’).

In the other districts, similar though less pronounced trends were seen (Fig. 2B and Supplementary Fig. 1); based on BAP denominators, Pacific people had the highest enrolment of all ethnic groups (although this never exceeded 100%), which was lowest based on HSU denominators. Unlike in Counties Manukau, the enrolled Māori population in other districts is increasing (Supplementary Fig. 1).

### Cervical cancer screening coverage

**Fig. 4:**
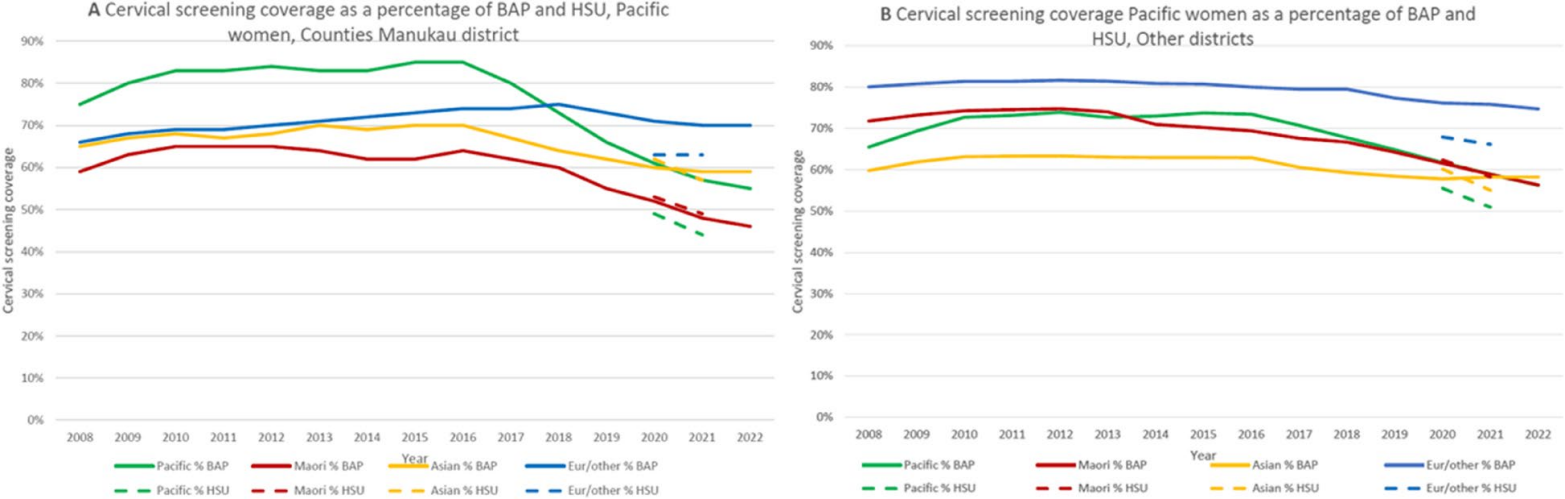
Trends in cervical screening coverage based on BAP and HSU denominators, respectively, by ethnic group in Counties Manukau (**4A**) and other Districts (**4B**), 2008–2022. BAP = Best Available Population, projections based on census data; Eur/Other = New Zealand European and other ethnic groups

Annual trends in cervical screening by ethnicity between 2008 and 2022 are shown in Fig. 4A (Counties Manukau District) and Fig. 4B (Other Districts), based on BAP [35] and HSU (Supplementary Table 1) denominators. For all ethnic groups, screening coverage gradually increased until 2016, when coverage started to decline for Māori, Pacific and Asian women, followed by European/Other women in 2018. Screening coverage for Pacific people in Counties Manukau was highest of all ethnic groups until 2018, when it dropped below that for the European/Other ethnic group. Replacing BAP with HSU denominators had the largest effect on Pacific estimates, that were now lowest of all ethnic groups, whereas Māori coverage slightly increased based on HSU.

**Fig. 5:**
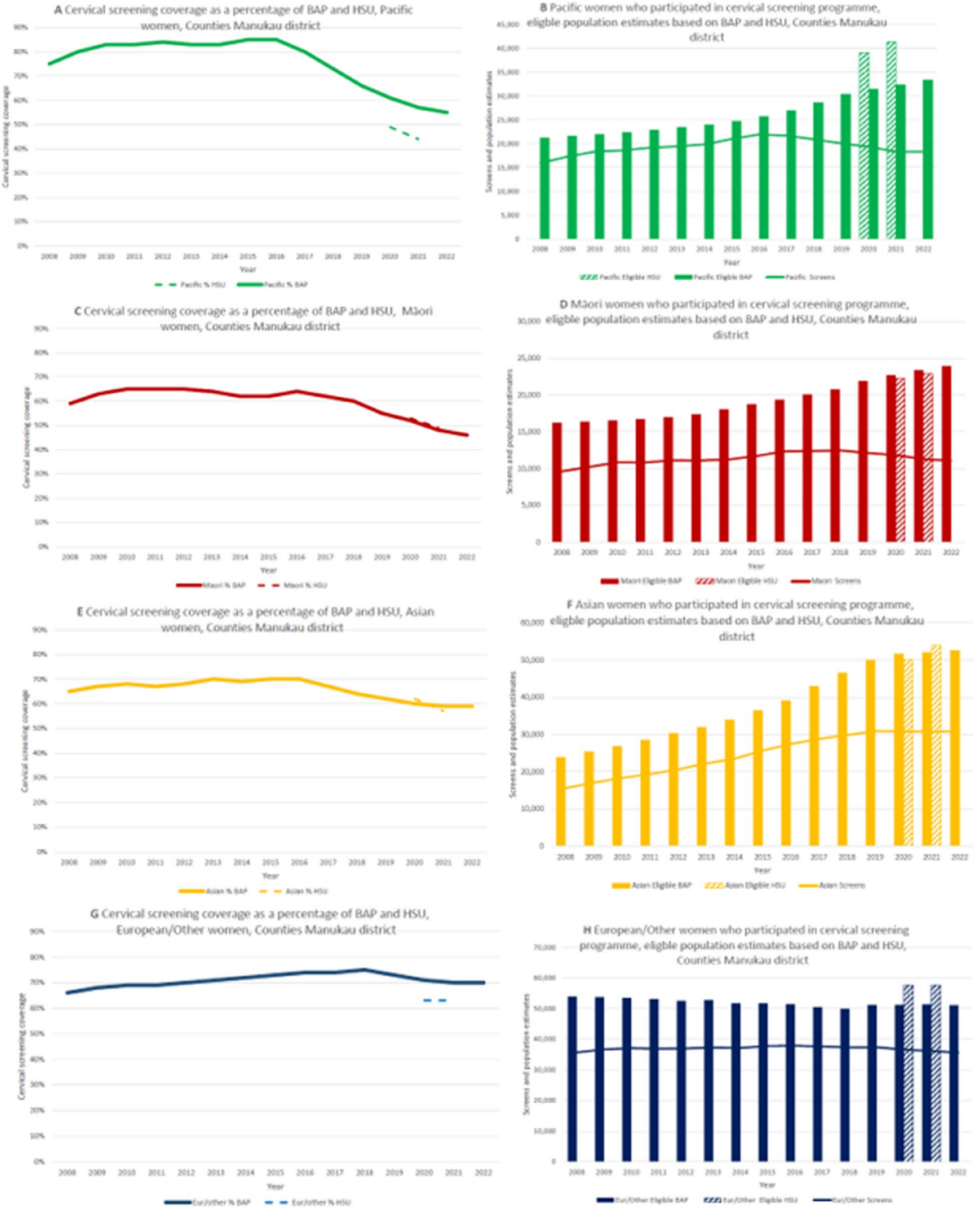
Trends in cervical screening coverage based on BAP and HSU denominators (**5A**, **5C**, **5E**) compared to trends in screened numbers (numerator) and estimated populations (denominators) (**4B**, **4D**, **4F**) by ethnic group in Counties Manukau District, 2008–2022. BAP = Best Available Population, projections based on census data; Eur/Other = New Zealand European and other ethnic groups

The numerators and denominators (Fig. 5) used for these estimates by ethnicity show that for Pacific women, screening numbers started to drop in 2016, followed by Māori in 2018, European/Other in 2019, whereas for Asian women it plateaued in 2019.

From 2017, there was a steady decline (15%) in the number of Pacific women screened (which contrasts with the increase in Pacific PHO enrolments in Counties Manukau), while the screening *coverage* dropped by 30%, based on adjustments in population projections.

In other districts, the same (but less pronounced) trends were observed (Supplementary Fig. 2).

## Discussion

Our study found substantial discrepancies when using BAP projections and HSU data for the denominator in health care statistics. These discrepancies were not equally distributed across geographic areas nor ethnic groups and were most pronounced in the Pacific population in South Auckland, where Pacific people do better than other ethnic groups with one denominator, and worse when using the other. It is important to understand that both denominators have specific and significant shortcomings.

The gold standard for official population statistics is the 5-yearly census and its derived population statistics. The infrequency of census data collection, the failure to require internal migrants to register changes in residential address [27], and the decentralisation of the health system into 20 health districts as part of the 2000 health reforms have created the need for more granular subnational population data. Ethnicity is frequently used in funding algorithms as it is an important determinant of health and health service use [33]. BAP has been developed for (health care) planning at subnational district level. Declining census participation (particularly for ethnic minority groups) inequitably affects not only the accuracy of census population size estimates, but also the quality of collected data. This affects the assumptions upon which BAP projections are modelled. Selective underestimates of the most vulnerable population groups lead to specific underfunding of these groups, and this contributes to increasing inequity in health.

Although population projections were never intended to be used as a denominator for health status statistics, BAP has increasingly been used for this purpose. Projected population statistics are based on population-level rather than individual data so cannot be linked to individual health statistics (as population-based registers can be). Health outcome statistics can only be ecologically compared with population projections at an aggregate level, resulting in ‘numerator-denominator’ discrepancies and biased results [31]. StatsNZ’s recommendation is to always validate the consistency of the numerator with the denominator in health statistics. This is not possible using BAP denominators, demonstrating that BAP data are not suitable for this purpose. The problems with the use of BAP are exposed when percentages over 100 occur.

In the absence of a population register, HSU data were used as a proxy to estimate COVID-19 vaccination coverages. This solved two problems: the numerator and denominator could be linked and percentages over 100 no longer occurred, and HSU data included addresses that are presumably updated more frequently than census address data and therefore are more accurate. This enabled estimating vaccination coverage by ethnicity, deprivation index and age group in any defined geographic area [40]. However, new, non-quantifiable biases were introduced. First, health user data are an inappropriate denominator for the uptake of health care, as underserved populations with no or limited health care access are invisible in the dataset. Furthermore, there are quality issues with ‘ethnicity’ and ‘address’ data in the HSU that disproportionately affect Māori and Pacific ethnic groups. Linking Census 2018 to HSU2020 data showed that 15–20% of people registered as Māori or Pacific in census data were not recorded as such in HSU [36]. Another study comparing HSU2018 to Census 2018 found 16% fewer Māori in HSU, and that Māori were more often misclassified than non-Māori ethnic groups [41]. A further problem for accurate Pacific health data is that HSU prioritises ethnicity data by Māori instead of reporting by total ethnicity, as recommended by StatsNZ. This under-represents Pacific people by another 15% [31].

Population undercounts can arise for several reasons, such as non-participation in censuses, under-use of civil services, under-use of health care, misclassification of data used to link datasets, immigrants who are not registered in birth and education registers, gaps in emigration data or linking problems with emigration data, etc. All these reasons can work in concert, and different reasons can apply to different ethnic groups.

Deprivation is related to geographic mobility and the number of changes in residential address increases with increasing deprivation [42]. Pacific (and Māori) people use health care less frequently and move more often, and therefore have less accurate address updates. Health user data are often used when address data are needed, for example for recalls for national vaccinations or cancer screening programmes. Inaccurate addresses and absence in health data contribute to disparities in health because people will not receive invitations for screening or recalls. Both disproportionately affect Māori and Pacific peoples [15].

BAP and HSU are not official statistics which means that the datasets are not regularly released or revised. Interim, unplanned adjustments can be made. BAP projections are preliminary and can be adjusted up to three years after publication [18]. The interim adjustments in both BAP and HSU after publication of the post-hoc review by StatsNZ [31] are likely to be the reason we could not reproduce the published reviewed data in our calculations. Unanticipated changes in methodology were also applied to Census 2018 (Table 1), which adds to the difficulty in reliably studying trends compared to data based on successive census statistics.

Systematic and consistent collection of data is essential to support the public health surveillance functions of monitoring trends over time, studying determinants for change, detecting (new) risk groups, and assessing the impact of interventions [43]. To be able to interpret trends reliably, stable denominators that accurately represent population changes are important. Most ANZ health statistics [35, 39, 44], including the national surveillance of notifiable diseases under the Health Act 1956 [45, 46] use BAP denominators. Most of these publications acknowledge that estimates are based on projections rather than actual statistics but do not clarify that the degree of inaccuracy differs between ethnic groups and between regions. Furthermore, projection methods can be changed at any time, including retrospectively up to 3 years after publication. In our trend evaluation of PHO enrolment and cervical cancer screening, we found that changes in trends mostly coincided with changes in population projections that had differing effects by ethnic group, rather than in changes in enrolment or screening numbers, making meaningful interpretation impossible.

Both indicator examples, ‘access to primary care’ and ‘uptake of cervical screening’, show that replacing the BAP denominator with HSU has different impacts on estimates in different geographic areas and on different ethnic groups, which cannot be corrected for. The denominator change did however consistently have the largest impact on ‘access to primary care’ and ‘uptake of cervical screening’ for the Pacific populations living in the neighbourhoods with the highest deprivation levels in the Counties Manukau district in South Auckland.

The indicators that are published on government websites and dashboards report that Pacific people have the *highest* coverage compared with all ethnic groups, and the coverage for Pacific people in Counties Manukau is higher than for Pacific people in other regions. It is more likely that these indicators for Pacific peoples are lowest of all ethnic groups, which is indeed what we found based on estimates using HSU denominators. Although the accuracy of HSU is unknown, it is safe to assume that HSU denominators are underestimates of the true population size, disproportionately so for smaller ethnic groups, making the real uptake for Pacific people even lower than the results we report based on the HSU denominator.

### The need for equitable denominators for health statistics: a real-time population register

Experiments are underway to investigate if collated national governmental administrative data in the IDI can contribute to or replace the census in the future [47, 48]. Combining administrative data has its own quality problems, largely based on non-uniform, inconsistent, and non-transparent methods for data collection [49]. In the current IDI, in theory, people can be simultaneously registered with as many different addresses and ethnicities as there are administrative databases. IDI deals with the problem of multiple datasets with different addresses and ethnicity by having a “personal details table” which ranks sources of data based on data quality assessments and includes variables such as “source ranked ethnicity”. Discrepancies between IDI and census cohorts were, again, found to be largest for people in the highest deprived neighbourhoods where most Māori and Pacific people live [47]. Complicated data cleaning procedures are in place which delays the availability of data and with suboptimal results [47]. It is unlikely that IDI data quality, which is collected by different agencies and not in controllable standardised ways will ever reach the standardised ethnicity data quality as collected on census night.

Under the current health reforms, it is intended to embed a population health approach in smaller locality areas than the previous health districts for better implementation of (public) health measures. Analysing data on a more granular level will magnify these data quality problems and further increase the inaccuracies.

A major improvement in the quality and completeness of population data could be achieved by developing an overarching, central governmental population register that collects ‘real-time’ basic demographic data. Instead of the current suite of ‘unique’ identifiers for health, tax, education etc., one national unique identifier would be introduced for each resident, replacing all other identifiers. When a person interacts with any governmental administrative service, the respective systems should retrieve the most up to date demographics from this central register, ask the person to check their details, correct where necessary, send the corrected data back to the register, update, and log the changes. Although the absence of a population register has been described as one of the main barriers in the transition to a census based on linked administrative registers, this major change towards a linked administrative census is now considered without addressing this barrier [50]. However, the creation of a comprehensive individual register would be an additional major change to the programme of official statistics, and would require the prior robust assessment of both social acceptability and the practicality of such a database of comprehensive routinely linked information.

Population-based registers that record all vaccinations administered to individuals targeted for vaccination *residing within a certain administrative area* is the gold standard to calculate vaccine coverage [51]. A recent review (2022) found that access to childhood vaccinations depends on enrolment in primary care, which is disproportionately delayed or hampered for Māori (and likely Pacific) children [52]. This contributes to the rapidly declining vaccination coverage in both ethnic groups. We recommended urgent action to develop a nationwide, centrally governed system that provides antenatal immunisations information, with a focus on vaccination coverage for groups with the lowest coverage. A 2020 review of the national cervical screening programme [53] also recommended developing a population-based register for ongoing audit and review of cervical cancer cases that utilise a consistent methodology and allow the selection of control groups for case control studies.

## Conclusion

BAP projections have not been developed for use as the denominator in health status statistics and are not suitable for this purpose. Replacing BAP with HSU as the denominator solved the numerator-denominator discrepancy, but this approach introduced new biases. Both denominators contribute to inequities in health status. Under the current health reforms, public health will be delivered in smaller geographic areas. The impact of using BAP denominators on the quality of health statistics will then be magnified. The focus on higher census coverage might give better population statistics and projections to use for health care planning, but it will not make BAP a suitable denominator for health statistics. The IDI has its own quality and equity issues and is therefore not a suitable denominator either. Investigating the practicality and social acceptability of a real-time population register is strongly recommended as a foundational tool to inform a more equitable health system. Meanwhile, reporting of ethnic specific outcomes and inequity needs to include a clear assessment of the potential for bias in measures based on inaccurate population counts and estimates.

### Supplementary Information

**Additional file 1: Supplementary Figure 1.** Trends in PHO enrolment percentage based on BAP and HSU denominators (S1A, S1C, S1E) compared to trends in enrolment (numerator) and estimated populations (denominators) (S1B, S1D, S1F) by ethnic group in ‘Other’ Districts, 2019-2022. **Supplementary Figure 2.** Trends in cervical screening coverage based on BAP and HSU denominators (S2A, S2C, S2E) compared to trends in screened numbers (numerator) and estimated populations (denominators) (S2B, S2D, S2F) by ethnic group in Counties Manukau District, 2008-2022.

**Additional file 2. **

## Data Availability

We used only publicly available data and refer to the websites.
