# Supplementary material for: Selective under-representation of Pacific peoples in population estimates for health indicator measurements in Aotearoa New Zealand misinforms policy making
Source: BMC Public Health. 2024 Feb 22;24:564. doi: 10.1186/s12889-024-17984-2 (PMC10882897; doi:10.1186/s12889-024-17984-2)
Supplement: Supplementary file 1 — Additional file 1: Supplementary Figure 1. Trends in PHO enrolment percentage based on BAP and HSU denominators (S1A, S1C, S1E) compared to trends in enrolment (numerator) and estimated populations (denominators) (S1B, S1D, S1F) by ethnic group in ‘Other’ Districts, 2019-2022. Supplementary Figure 2. Trends in cervical screening coverage based on BAP and HSU denominators (S2A, S2C, S2E) compared to trends in screened numbers (numerator) and estimated populations (denominators) (S2B, S2D, S2F) by ethnic group in Counties Manukau District, 2008-2022. [file 12889_2024_17984_MOESM1_ESM.docx]

Supplementary Figure 1. Trends in PHO enrolment percentage based on BAP and HSU denominators (S1A, S1C, S1E) compared to trends in enrolment (numerator) and estimated populations (denominators) (S1B, S1D, S1F) by ethnic group in ‘Other’ Districts, 2019-2022.

.


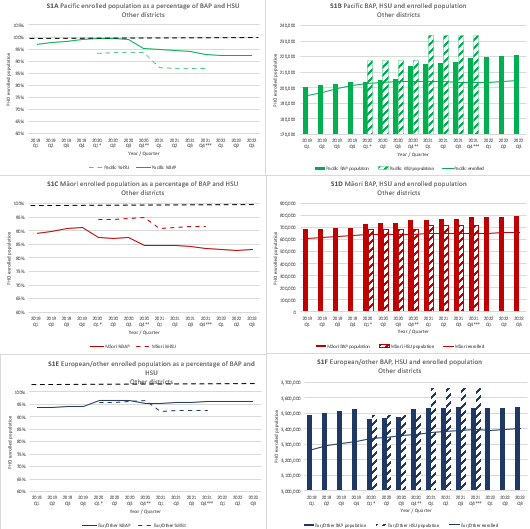


PHO = Primary Healthcare Organisation; BAP = Best Available Population, projections based on census data; Q = quarter; Eur/Other = New Zealand European and other ethnic groups

- - - - - - 100% line

*Interim adjustment in BAP. The updated projections use the latest subnational population estimates and updated methodology to calculate migration. The combined impact is a large variation in projected population for some DHBs when comparing to previous releases and this is most noticeable in Auckland.

**Interim adjustment in BAP. Population is based on projections provided by Stats NZ in Dec 2020.

***Interim adjustment in BAP. Population is based on projections provided by Stats NZ in Dec 2021.

Supplementary Figure 2. Trends in cervical screening coverage based on BAP and HSU denominators (S2A, S2C, S2E) compared to trends in screened numbers (numerator) and estimated populations (denominators) (S2B, S2D, S2F) by ethnic group in Counties Manukau District, 2008-2022.


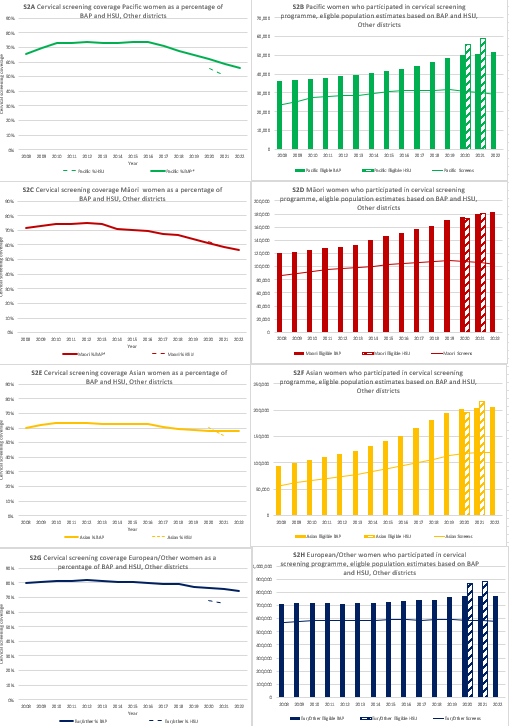


BAP = Best Available Population, projections based on census data; Eur/Other = New Zealand European and other ethnic groups
